# Supplementary material for: Assessment of severity scoring systems for predicting mortality in critically ill patients receiving continuous renal replacement therapy
Source: PLoS One. 2023 May 25;18(5):e0286246. doi: 10.1371/journal.pone.0286246 (PMC10212150; doi:10.1371/journal.pone.0286246)
Supplement: Attachment — Submitted filename: (20230308) Response to reviewers.docx [file pone.0286246.s001.docx]

**Response to reviewer(s)’ comments**

Dear Reviewer(s),

We are grateful for the valuable comments. After thorough discussions, we have revised our paper to reflect your helpful recommendations. Our point-by-point responses to the comments appear in blue in the following, and the revised manuscript can be found enclosed with the submission.

**Reviewer 1**
**The study by Chun et al is an original research to attempt using well validated scoring systems to predict 7 day and in hospital mortality in patients on CRRT. The study does follow the necessary statistical requirement to compare scoring systems and uses the standard metrics to judge prognostic scoring systems including discrimination and calibration. Unfortunately the conclusions are not valid since there are methodological flaws. Firstly many of the scores being compared have used in their developmental cohort patients only with AKI needing CRRT unlike authors study where CKD patients needing dialysis is also included. Additionally, they do not mention what percentage of their patients had CKD among survivors vs non-survivors.**

- Thank you for your thorough comments. Many studies included all patients diagnosed with AKI who received renal replacement therapy such intermittent hemodialysis. Since our study focused on CRRT, we included CKD patients who received CRRT, except for those who received renal replacement therapy such as intermittent hemodialysis, peritoneal dialysis, or kidney transplants. As you mentioned, we added proportion of CKD patients as following:

**(Line 127-129)** CKD patients accounted for 11.9% of the total study population, and there was no significant difference in the proportion of survivors and non-survivors.

**(Table 1: Line 134-138)**

**Table 1. Demographics and Clinical Characteristics of Study Population**

| Parameter | All patients  n=612 (%) | Survivors  n=167 (%) | Non-survivors  n=445 (%) | P-value |
| --- | --- | --- | --- | --- |
| Age(years) | 67.6 ± 14.8 | 67.4 ± 14.9 | 67.7 ± 14.7 | 0.851^*^ |
| Sex |  |  |  |  |
| Male | 365 (59.6) | 95 (56.9) | 270 (60.7) | 0.406^+^ |
| Female | 247 (40.4) | 72 (43.1) | 175 (39.3) |  |
| Etiology of AKI |  |  |  |  |
| Sepsis | 400 (65.4) | 108 (64.7) | 292 (65.6) | 0.849^+^ |
| Nephrotoxic | 226 (36.9) | 54 (32.3) | 172 (38.7) | 0.159^+^ |
| Ischemic | 204 (33.3) | 52 (31.1) | 152 (34.2) | 0.502^+^ |
| Others | 62 (10.1) | 18 (10.8) | 44 (9.9) | 0.764^+^ |
| CKD history | 73 (11.9) | 24 (11.4) | 49 (11.0) | 0.264 |
| Comorbidities |  |  |  |  |
| Diabetes Mellitus | 231 (37.7) | 81 (48.5) | 150 (33.7) | 0.001^+^ |
| Hypertension | 336 (54.9) | 99 (59.3) | 237 (53.3) | 0.202^+^ |
| Heart Failure | 91 (14.9) | 27 (16.2) | 64 (14.4) | 0.610^+^ |
| Coronary Artery Disease | 94 (15.4) | 24 (14.4) | 70 (15.7) | 0.708^+^ |
| COPD | 13 (2.1) | 2 (1.2) | 11 (2.5) | 0.530^+^ |
| Cancer | 111 (18.1) | 21 (12.6) | 90 (20.2) | 0.034^+^ |
| Liver disease | 100 (16.3) | 18 (10.8) | 82 (18.4) | 0.027^+^ |

Continuous data are presented as mean ± SD, and categorical data as number of patients (%). AKI, acute kidney injury; COPD, chronic obstructive pulmonary disease.

*P-value by Student's t-test

+P-value by Fisher's exact test

**Besides this there is no mention of timing of scoring with respect to CRRT start.**

- Following your recommendation, we added the timing of scoring as following:

**(Line 109-111)** Severity scoring was performed when it was decided to start CRRT, and all variables for severity scores were collected within 24 hours prior to the initiation of CRRT.

**Minor issues: Sentences need to structured for more clarity: for instance**

**Line 71 : which study is mentioned here**

**Line 72: Another several studies is grammatically incorrect. I think authors want to say that other studies have compared predictive power of disease severity scoring systems**

**Line 74 : needs clarification**

- We appreciate for your help. We rephrased the sentences as the following:

**(Line 68-70)** In addition, in studies to improve the quality of CRRT, such as the optimal start time of CRRT [25, 26],

**(Line 71-72)** Other studies have compared predictive power of severity scoring systems in patients with AKI.

**(Line 72-74)** However, many studies included all patients diagnosed with AKI who received renal replacement therapy such intermittent hemodialysis, and few studies focused solely on CRRT [28-34].

**Reviewer 2**
**1. The authors overstate how good any of these models are. For example, in the results the authors state that, "The AUROC revealed high discrimination ability of Demirjian’s score followed by Liano's score." In the discussion, they state that, "The AUROC for in-hospital mortality revealed high discrimination ability of Demirjian’s score (0.770) followed by Liano's score (0.728) and APACHE II score (0.710)." I would not agree with these statements. Though it's somewhat arbitrary, most would consider AUROC of 0.7-0.8 to have "moderate" or "acceptable" predictive ability. For example, a frequently cited interpretation of AUROC is as follows:**

**0.5 = No discrimination**

**0.5-0.7 = Poor discrimination**

**0.7-0.8 = Acceptable discrimination**

**0.8-0.9= Excellent discrimination**

**>0.9 = Outstanding discrimination**

**[From: Hosmer, D.W., Jr., Lemeshow, S. and Sturdivant, R.X. (2013). Assessing the Fit of the Model. In Applied Logistic Regression (eds D.W. Hosmer, S. Lemeshow and R.X. Sturdivant). https://doi.org/10.1002/9781118548387.ch5]**

**Overall, the results of this study are disappointing -- only 1 test was had borderline good AUC (Demirjian’s), but even that test had poor fit. I think the authors needs to do a better job of explaining that none of these tests are particularly good, and therefore they should more strongly emphasize that the primary conclusion of this study is that additional research is needed to develop better scoring systems.**

- Thank you for your careful review of our work and your suggestions regarding the manuscript. Following your recommendation, we rephrased the sentences and added the opinion in the conclusion as the following:

**(Line 163-165)** The AUROC revealed acceptable discrimination ability of Demirjian’s score followed by Liano's score.

**(Line 183-184)** Same as in-hospital mortality, Demirjian’s score showed relatively high value to predict the 7-day mortality.

**(Line 201-202)** The AUROC for in-hospital mortality revealed acceptable discrimination ability of Demirjian’s score

**(Line 285-289)** However, none of those evaluated in this study showed both excellent differentiation and suitability. In conclusion, all severity scoring systems included in this study were inappropriate for predicting mortality of critically ill patients requiring CRRT. Therefore, we emphasize the need to develop a novel severity scores with good calibration and high discrimination for the patients.

**2. Why was the mortality of the cohort so high? The authors should try to better address this. The authors suggest that AKI treated specifically with CRRT has higher mortality than AKI patients overall, which is true, but even for AKI requiring CRRT this is high. In most studies, the mortality of AKI requiring CRRT is closer to 50%. Comparing it to the BEST study (reference 6) -- which reported data from patients treated >20 years -- doesn't seem to adequate address the extremely high mortality seen here. To the degree that these authors are looking to evaluate mortality prediction tools, analyzing a cohort that has a much higher mortality than other centers could make the findings less generalizable. To help address this, they should report the mean or median and distribution of the disease severity scores of this cohort, particularly those that are a reflection of overall disease severity (i.e., SOFA, SAPS 3, APACHE 2, MODS). Regardless of whether they are able to somehow justify such a high mortality, this high mortality should be included as a potential limitation of the study, because these results may not be as relevant to centers with different patient selection and/or better CRRT outcomes.**

- Thank you for your help. We added the mean severity scores of the study population. Also we added that high mortality is a potential limitation of our study as the following:

**(Line 149-152)** The mean severity scores of the study population were APACHE II score 35.5, SAPS 3 84.6, SOFA score 9.0, MODS 10.7, Liano's score 0.55, PICARD score 0.43, and Demirjian's score 0.60. Table 3 shows the comparison of severity score between survivors and non-survivors. There was significant difference in the mean of all severity scores between survivors and non-survivors (all p <0.01).

**(Table 3: Line 154-159)**

**Table 3. Comparison of severity score between survivors and non-survivors**

| Severity score | All patients  (n=612)  mean±SD | Survivors  (n=167)  mean±SD | Non-survivors  (n=445)  mean±SD | P-value |
| --- | --- | --- | --- | --- |
| APACHE II score | 35.5 ± 9.3 | 30.6 ± 8.8 | 37.3 ± 8.8 | <0.001^*^ |
| SAPS 3 | 84.6 ± 19.9 | 73.6 ± 17.7 | 87.7 ± 19.8 | <0.001^*^ |
| SOFA score | 9.0 ± 3.3 | 8.0 ± 3.3 | 9.4 ± 3.2 | <0.001^*^ |
| MODS | 10.7 ± 4.3 | 9.8 ± 0.5 | 11.1 ± 0.1 | 0.001^*^ |
| Liano's score | 0.55 ± 0.22 | 0.42 ± 0.19 | 0.60 ± 0.21 | <0.001^*^ |
| PICARD score | 0.43 ± 0.16 | 0.37 ± 0.15 | 0.46 ± 0.15 | <0.001^*^ |
| Demirjian's score | 0.60 ± 0.31 | 0.38 ± 0.29 | 0.68 ± 0.28 | <0.001^**^ |

Data are presented as mean ± SD. APACHE, Acute Physiology and Chronic Health Evaluation; SAPS, Simplified Acute Physiology Score; SOFA, Sequential Organ Failure Assessment; MODS, Multiple Organ Dysfunction Score; PICARD, The Program to Improve Care in Acute Renal Disease.

*P-value by Student's t-test

**P-value by Mann-Whitney test

**(Line 216-218)** Despite this reason, the mortality rate of our study population, which is relatively higher than that of other studies, is a potential limitation of this study.

**3. This is extremely poorly worded: "Except for Liano's score (χ2=7.555, 166 p=0.478) and PICARD score (χ2=14.835, p=0.062), the Hosmer-Lemeshow test for in-hospital mortality demonstrated that calibration ability of all scores was not significant." Essentially, the authors are misusing the word significant here. The term "significant" when describing a statistical test indicates you reject the null hypothesis because the observed findings are unlikely to be due to change (i.e., p is <0.05). Technically, for Liano and PICARD in this study, the results of the Hosmer-Lemeshow test are *not* statistically significant (i.e., the null hypothesis that the predicted and observed outcomes are the same is not rejected). For Hosmer-Lemeshow test, a significant test indicates that the model is *not* a good fit, and a non-significant test indicates a good fit. I would suggest the authors report the results of the Hosmer-Lemeshow tests by simply providing the appropriate interpretation and avoiding the terms "significant" or "not significant", which are confusing in general for this type of test. For example, the authors could state, "Except for Liano's score (χ2=7.555, 166 p=0.478) and PICARD score (χ2=14.835, p=0.062), the Hosmer-Lemeshow test for in-hospital mortality demonstrated poor fit of the prediction models (p <0.05)." Similarly, this statement, "The APACHE II score was more discriminative compared to other general scores, but the results of the fitness test were not significant" could be changed to "The APACHE II score was more discriminative compared to other general scores, but the results of the fitness test showed poor calibration." Similarly, the abstract should state, "The Hosmer-Lemeshow test demonstrated good fit of Liano's score and PICARD scores." Likely, in the discussion I suggest, "The Hosmer-Lemeshow test on Demirjian’s score showed poor fit in our analysis, but it was more acceptable compared to the general severity scores."**

- We appreciate for your help. We rephrased the sentences as the following:

**(Line 189-190)** Except for Liano's score (χ2=7.555, p=0.478) and PICARD score (χ2=14.835, p=0.062), the Hosmer-Lemeshow test for in-hospital mortality demonstrated poor fit of the prediction models (p <0.05).

**(Line 206-207)** In addition, the Hosmer-Lemeshow test results of five scores, which provide predicted mortality, showed poor calibration of all scores except Liano's and PICARD scores.

**(Line 220-221)** The APACHE II score was more discriminative compared to other general scores, but the results of the fitness test showed poor calibration,

**(Line 40-42)** The Hosmer-Lemeshow test on Demirjian’s score showed poor fit in our analysis, but it was more acceptable compared to the general severity scores.

**4. Why are the authors publishing retrospective data from 2016-2018 in 2023? That seems odd. It certainly takes time to do this research, but a 4-year interval seems much. Is this a secondary analysis of a cohort which was generated for a prior study? If so, the authors should cite the prior work. Otherwise, at least a brief explanation as to why these patients from a cohort that is >4y old at publication seems warranted.**

- We agree with your opinion. The reason we used the data from 2016 to 2018 is that the data at that time was accessible. Since the author belonged to the department at the time, it was easy to access the data at the time. After data collection, an actual analysis was conducted from 2019 to 2020. Above all, the authors believe that the characteristics of the cohort in this study, which evaluates the adequacy of scoring systems for a particular group, will not change the relevant risk factors of the study results.

**5. Table 3, as written, is somewhat confusing. It is hard to follow the directionality of comparisons. To help make it easier to follow, I suggest putting the score with the highest AUC first (Demirjian), followed by the second highest (Liano), etc. That should make it a little easier to read.**

- Thank you for your thorough comments. As you mentioned, we rephrased the table as the following:

**(Table 4: Line 171-173)**

Table 4. Pairwise Comparison of Receiver Operating Characteristic Curves for In-hospital Mortality

| Severity score system | | Compared scores | | 95% CI | P-value |
| --- | --- | --- | --- | --- | --- |
| Demirjian score | ~ | | Liano score | -0.005 - 0.091 | 0.081 |
|  | ~ | | APACHE II score | 0.009 - 0.111 | 0.020 |
|  | ~ | | SAPS 3 | 0.050 - 0.149 | <0.001 |
|  | ~ | | MODS | 0.053 - 0.157 | <0.001 |
|  | ~ | | PICARD score | 0.065 - 0.159 | <0.001 |
|  | ~ | | SOFA score | 0.093 - 0.204 | <0.001 |
| Liano score | ~ | | APACHE II score | -0.029 - 0.063 | 0.459 |
|  | ~ | | SAPS 3 | 0.008 - 0.106 | 0.023 |
|  | ~ | | MODS | 0.004 - 0.121 | 0.036 |
|  | ~ | | PICARD score | 0.006 - 0.132 | 0.031 |
|  | ~ | | SOFA score | 0.042 - 0.017 | 0.001 |
| APACHE II score | ~ | | SAPS 3 | -0.002 - 0.082 | 0.064 |
|  | ~ | | MODS | -0.005 - 0.095 | 0.076 |
|  | ~ | | PICARD score | -0.011 - 0.115 | 0.105 |
|  | ~ | | SOFA score | 0.028 - 0.148 | 0.004 |
| SAPS 3 | ~ | | MODS | -0.042 - 0.054 | 0.816 |
|  | ~ | | PICARD score | -0.044 - 0.069 | 0.664 |
|  | ~ | | SOFA score | -0.005 - 0.102 | 0.073 |
| MODS | ~ | | PICARD score | -0.053 - 0.067 | 0.825 |
|  | ~ | | SOFA score | 0.001 - 0.086 | 0.048 |
| PICARD score | ~ | | SOFA score | -0.025 - 0.097 | 0.241 |

**6. In the introduction, I found this statement to be somewhat misleading: "This is because the therapy is hemodynamically more stable than the intermittent hemodialysis therapy, and it is easy to control fluid balance and to correct metabolic acidosis or electrolyte imbalance and to correct nutritional deficiency [9]." In general, intermittent HD corrects acidosis and electrolytes just as well as CRRT. I also don't understand what is meant by "correct nutritional deficiency". I would simply end the sentence after "...control fluid balance." If they want to claim that CRRT is better for acidosis or electrolytes, a much more complicated discussion about instantaneous clearance vs. today daily dose of RRT (i.e., equilibrated Kt/V) would be needed, but it’s just best to avoid suggesting that CRRT is better than IHD for acidosis or electrolytes.**

- We appreciate your suggestion. We rephrased the sentence as the following:

**(Line 54-55)** This is because the therapy is hemodynamically more stable than the intermittent hemodialysis therapy, and it is easy to control fluid balance [9].

**7. For the calibration tests, why did the author present results for only 5 of the 7 scoring systems? They should present them all or explain why they excluded SOFA and MODS.**

- We added the reasons for exclusion as following:

**(Line 187-188)** Two severity scoring systems were excluded, SOFA score and MODS, which do not generate probability of death, only counting points.

**8. In the discussion, for all the other prediction scores used (e.g., Mehta, Chertow, Paganini, SHARF II, and VELLORE), the authors should cite the original publications describing these scoring systems.**

- Additional references is added as following:

**(Line 235-236)** Mehta score [35], Liano's score, Chertow score [36]

**(Line 240)** Logistic Organ Dysfunction [38], Organ Dysfunction and Infection [39]

**(Line 246)** SHARF II score [40], PICARD score, VELLORE score [41],

**(Line 384-402)**

35.	Mehta RL, Pascual MT, Gruta CG, Zhuang S, Chertow GM. Refining predictive models in critically ill patients with acute renal failure. J Am Soc Nephrol. 2002; 13:1350–7.

36.	Chertow GM, Lazarus JM, Paganini EP, Allgren RL, Lafayette RA, Sayegh MH. Predictors of mortality and the provision of dialysis in patients with acute tubular necrosis. The Auriculin Anaritide Acute Renal Failure Study Group. J Am Soc Nephrol. 1998;9:692–8.

37.	Paganini EP, Halstenberg WK, Goormastic M. Risk modeling in acute renal failure requiring dialysis: The introduction of a new model. Clin Nephrol. 1996; 46:206–11.

38.	Le Gall JR, Klar J, Lemesho S, Saulnier F, Alberti C, Artigas A, et al. The Logistic Organ Dysfunction System. A New Way to Assess Organ Dysfunction in the Intensive Care Unit. JAMA. 1996;276(10):802-10.

39.	Fagon JY, Chastre J, Novara A, Medioni P, Gibert C. Characterization of intensive care unit patients using a model based on the presence or absence of organ dysfunctions and/or infection: The ODIN model. Intensive Care Medicine. 1993;19:137-44.

40.	Lins RL, Elseviers MM, Daelemans R, Arnouts P, Billiouw JM, Couttenye M, et al. Re-evaluation and modification of the stuivenberg hospital acute renal failure (SHARF) scoring system for the prognosis of acute renal failure: an independent multicentre, prospective study. Nephrol Dial Transplant. 2004;19:2282–8.

41.	Dharan KS, John GT, Antonisamy B, Kirubakaran MG, Jacob CK. Prediction of mortality in acute renal failure in the tropics. Ren Fail. 2005;27:289–96.

**9. Do the authors have the distribution of baseline creatinine or eGFR? Or do they have the proportion of patients with advanced (e.g., stage 4 or 5) CKD? The authors suggest some patients with advanced CKD were included in the cohort. These patients (which many of which may be better classified as new ESKD rather than AKI) could be vastly different than patients with AKI. To better understand this cohort, more information about the relative number of patients with de novo AKI, AKI on CKD, and progressive CKD would be good.**

- Thank you for your careful comments. We provided the proportion of CKD patients regarding your comments. We also added this characteristic of the cohort as a limitation of our study as following:

**(Line 127-129)** CKD patients accounted for 11.9% of the total study population, and there was no significant difference in the proportion of survivors and non-survivors.

**(Table 1: Line 134-139)**

**Table 1. Demographics and Clinical Characteristics of Study Population**

| Parameter | All patients  n=612 (%) | Survivors  n=167 (%) | Non-survivors  n=445 (%) | P-value |
| --- | --- | --- | --- | --- |
| Age(years) | 67.6 ± 14.8 | 67.4 ± 14.9 | 67.7 ± 14.7 | 0.851^*^ |
| Sex |  |  |  |  |
| Male | 365 (59.6) | 95 (56.9) | 270 (60.7) | 0.406^+^ |
| Female | 247 (40.4) | 72 (43.1) | 175 (39.3) |  |
| Etiology of AKI |  |  |  |  |
| Sepsis | 400 (65.4) | 108 (64.7) | 292 (65.6) | 0.849^+^ |
| Nephrotoxic | 226 (36.9) | 54 (32.3) | 172 (38.7) | 0.159^+^ |
| Ischemic | 204 (33.3) | 52 (31.1) | 152 (34.2) | 0.502^+^ |
| Others | 62 (10.1) | 18 (10.8) | 44 (9.9) | 0.764^+^ |
| CKD history | 73 (11.9) | 24 (11.4) | 49 (11.0) | 0.264 |
| Comorbidities |  |  |  |  |
| Diabetes Mellitus | 231 (37.7) | 81 (48.5) | 150 (33.7) | 0.001^+^ |
| Hypertension | 336 (54.9) | 99 (59.3) | 237 (53.3) | 0.202^+^ |
| Heart Failure | 91 (14.9) | 27 (16.2) | 64 (14.4) | 0.610^+^ |
| Coronary Artery Disease | 94 (15.4) | 24 (14.4) | 70 (15.7) | 0.708^+^ |
| COPD | 13 (2.1) | 2 (1.2) | 11 (2.5) | 0.530^+^ |
| Cancer | 111 (18.1) | 21 (12.6) | 90 (20.2) | 0.034^+^ |
| Liver disease | 100 (16.3) | 18 (10.8) | 82 (18.4) | 0.027^+^ |

Continuous data are presented as mean ± SD, and categorical data as number of patients (%). AKI, acute kidney injury; COPD, chronic obstructive pulmonary disease.

*P-value by Student's t-test

+P-value by Fisher's exact test

**(Line 277-281)** In this study, CKD patients were included in the study population, except for patients who received renal replacement therapy such as intermittent hemodialysis, peritoneal dialysis, and kidney transplantation. Since the focus is on patients receiving CRRT, the study results are unlikely to change due to the characteristics of this cohort, but the lack of information such as baseline creatinine or eGFR is a limitation of this study**.**
