# Supplementary material for: Assessment of severity scoring systems for predicting mortality in critically ill patients receiving continuous renal replacement therapy
Source: PLoS One. 2023 May 25;18(5):e0286246. doi: 10.1371/journal.pone.0286246 (PMC10212150; doi:10.1371/journal.pone.0286246)
Supplement: Attachment — Submitted filename: (20230422)_Response to reviewers.docx [file pone.0286246.s002.docx]

**Response to reviewer(s)’ comments**

Dear Reviewers (s):

We are grateful for your insightful comments. After thorough discussions, we have revised our paper to reflect your valuable recommendations. Our point-by-point responses to the comments are provided in blue in the following text, and the revised manuscript is enclosed with the submission.

**Reviewer 2**
**1. In the discussion [lines 263-265 in the clean version] I once again suggest, "The Hosmer-Lemeshow test on Demirjian’s score showed poor fit in our analysis, but it was more acceptable compared to the general severity scores."**

Response: Thank you for your careful review of our work and for your suggestions regarding this manuscript. Following your recommendation, we have rephrased the sentence as follows:

**(Line 270-271)** The Hosmer-Lemeshow test on Demirjian’s score showed a poor fit in our analysis; however, it was more acceptable than general severity scores.

**2. The term suitability is vague. In the conclusions, I recommend changing, "However, none of those evaluated in this study showed both excellent differentiation and suitability" to a more precise statement, "However, none of those evaluated in this study showed both excellent differentiation and calibration."**

Response: Thank you for your insightful comment. We have rephrased the sentence as follows:

**(Line 292-293)** However, none of the parameters evaluated in this study exhibited both excellent differentiation and calibration.

**3. In the conclusion I also suggest being a bit less extreme by stating, "In conclusion, all severity scoring systems included in this study had limited ability to predict mortality of critically ill patients requiring CRRT."**

Response: Thank you for your valuable comments. As per your suggestion, we have rephrased the sentence as follows:

**(Line 294-295)** In conclusion, all severity scoring systems included in this study had a limited ability to predict mortality in critically ill patients requiring CRRT.

**4. As I commented in my first review, I think the manuscript could significantly benefit from additional English language editing.**

Response: Thank you for the valuable suggestion. We would like to thank Editage (www.editage.co.kr) for reviewing and editing the revised manuscript for English language.
